# Engagement and attrition in digital mental health: current challenges and potential solutions

**DOI:** 10.1038/s41746-025-01778-w

**Published:** 2025-07-02

**Authors:** Katharine A. Smith, Thomas Ward, Sinéad Lambe, Edoardo G. Ostinelli, Charlotte Blease, Thomas Gant, Stefan M. Gold, Emily A. Holmes, Ivana Paccoud, Anastasia Vinnikova, Jochen Klucken, Peter J. Uhlhaas, Carolina Garcia Sanchez, Kate Haining, Kerem Böge, Sofiia Lahutina, Luisa Tomelleri, Sean Ryan, John Torous, Andrea Cipriani

**Affiliations:** 1 Department of Psychiatry, University of Oxford, Oxford, UK; 2 Oxford Health NHS Foundation Trust, Warneford Hospital, Oxford, UK; 3 Oxford Precision Psychiatry Lab, NIHR Oxford Health Biomedical Research Centre, Oxford, UK; 4 Department of Psychology, Institute of Psychiatry, Psychology and Neuroscience, King’s College London, London, UK; 5 South London & Maudsley NHS Foundation trust, London, UK; 6 Department of Experimental Psychology, University of Oxford, Oxford, UK; 7 Department of Women’s and Children’s Health, Uppsala University, Uppsala, Uppsala County Sweden; 8 Charité – Universitätsmedizin Berlin, Dept Psychiatry and Dept Psychosomatic Medicine, Campus Benjamin Franklin, Berlin, Germany; 9 German Center for Mental Health (DZPG), Partner Site Berlin/Potsdam, Berlin, Germany; 10 INIMS, Universitätsklinikum Hamburg-Eppendorf, Hamburg, Germany; 11 Luxembourg Centre for Systems Biomedicine (LCSB), University of Luxembourg, Esch-sur-Alzette, Luxembourg; 12 Independent researcher (Patient and Public Involvement and Engagement (PPIE) representative), London, UK; 13 Centre Hospitalier de Luxembourg (CHL), Luxembourg, Luxembourg; 14 Dept. of Child and Adolescent Psychiatry, Psychosomatic Medicine and Psychotherapy, Charité – Universitätsmedizin, Berlin, Germany; 15 School of Psychology and Neuroscience, University of Glasgow, Glasgow, UK; 16 German National Academy of Sciences Leopoldina, Halle, Germany; 17 Centrum für Affektive Neurowissenschaften, Charité - Universitätsmedizin Berlin, Berlin, Germany; 18 Santa Giuliana Hospital, Verona, Italy; 19 Division of Digital Psychiatry, Beth Israel Deaconess Medical Center, Harvard Medical School, Boston, MA USA

**Keywords:** Neurological disorders, Psychiatric disorders, Patient education, Outcomes research

## Abstract

In digital mental health engagement rates are consistently low, which may limit its effects. Using an international multidisciplinary consensus method, including lived experience expertise and a systematic review, we identified three key challenges: (i) lack of agreed metrics for engagement; (ii) lack of evidence on how better engagement improves outcomes; (iii) lack of standards for user involvement. Three potential solutions encompassed: (i) standardisation of frameworks for reporting engagement metrics and optimal doses of digital tools, (ii) measuring engagement with more precise reporting of outcomes, including potential harms; (iii) defining standards of user involvement (including appropriate diversity, and clinician as well as user input). Digital interventions have real potential in meeting the shortfall in service provision for mental health, but this will require focus on high quality research studies of the underlying mechanisms of engagement and optimal outcomes. Our findings identify and highlight the next best steps in this process.

## Introduction

Engagement is an essential element for any digital health tool^1^. However, user engagement with digital mental health interventions (DMHIs) as assessed by usage data remains consistently low^2^. This presents a fundamental challenge: *how can tools designed to support mental health succeed if users do not actively use or engage with them?*

Digital interventions provide an unprecedented opportunity to study the usage aspects of engagement; unlike traditional healthcare settings where engagement can be challenging to quantify, digital tools can generate real-time, automated usage data such as logins, pageviews, time used and even eye gaze that offer valuable insights into user behaviour^3^. However, engagement involves more than this, as usage data alone does not capture the degree of investment of the participant in the activity^4^. Thus, many digital health studies have also used qualitative methods such as questionnaires or semi-structured interviews to help capture some of the more nuanced and complex aspects of engagement, including satisfaction, acceptability and usability. Despite these efforts, there has been huge variability in the terminology applied to both usage and self-report measures^5,6^. Although several conceptual frameworks for engagement have been proposed^7^, capturing the complex construct of meaningful engagement, including not only usage, but also its cognitive, emotional and behavioural dimensions, has proved challenging^8^. The variation in definitions has also impeded meaningful comparisons of metrics of engagement across studies^6^ and also across assessments of outcomes, because measured outcomes may underestimate the intervention treatment effects when engagement has been poor or variably reported^3^.

Given these challenges in such a key area in digital health, we implemented a novel approach: we used a well-established consensus methodology, with international multidisciplinary expertise including lived experience, and incorporated a systematic search of the evidence to identify both the challenges and the potential solutions to guide the study and optimisation of effective engagement in DMHIs. We also considered how this might translate to improved outcomes for users.

## Results

The consensus group identified three broad areas of challenge in understanding engagement in DMHIs, which are summarised in Table 1.

**Table 1:** Areas of challenge in assessing engagement and adherence in digital studies in brain health (as identified by the consensus group)

**1. Lack of universally agreed definitions of metrics related to engagement** • Terms such as usage, engagement, adherence, attrition, and empowerment all have variable definitions in individual studies • Raw data are often not reported
**2. Lack of evidence of how or whether improved engagement improves outcomes**. • No clear evidence that increased engagement improves outcomes • No clear evidence of a dose effect of engagement or the optimal dose needed • Difficulties of translating engagement from the research to real-world settings • No clear evidence on the exact mechanisms of engagement, individual effective use patterns and attrition • Interaction with mental health • Attrition • Lack of adverse event reporting
**3. User involvement in developing and delivering digital health interventions** • User involvement can occur in different ways and levels of intensity and inclusivity • Reporting of user involvement is variable • User centred design may improve engagement and outcomes, but more direct evidence is needed

### Definitions of metrics related to engagement

The consensus group agreed on the lack of clarity across studies and the lack of universally agreed, standardised definitions of the metrics related to engagement^3,8^. Terms such as usage, adherence, engagement, and attrition are used to describe aspects of engagement, and empowerment to describe patient related outcomes of engagement, but they often have overlapping definitions and vary between individual studies. For example, ‘engagement’ is often equated directly to the frequency or duration of usage but their definitions vary between studies^3,5,9^. In addition, there was consensus that studies often do not report the raw data needed to make comparisons between studies. For example, in a scoping review focussing on DMHIs for depression, only 59% (13/22) of studies reported usage statistics^10^. Within usage statistics themselves, studies often do not report the original data but instead a measure of ‘adherence’, usually an assessment of compliance with a pre-specified metric of completion (e.g., a set number of modules). Some definitions of adherence are more nuanced and incorporate not only usage and intended use but also justification for how intended use was defined^11^; however, definitions vary and often lack a clear rationale for their selection. Even in studies where usage or adherence are reported, this does not necessarily reflect engagement, which is a more complex concept involving not only usage but also cognitive, affective and motivational components^12^. The individual concepts are related (for example, usage is a pre-requisite for engagement and adherence), but they differ in scope and complexity.

Despite these challenges in definition, the consensus group agreed that engagement remains a key concept for study in DMHIs as this is an essential first step in effecting positive outcomes. Digital interventions provide a unique opportunity to investigate the mechanisms of engagement through their ability to automatically capture detailed data on usage patterns, user interactions, and associated outcomes^3^ and identify interventions that can maximize effective engagement and positive outcomes.

### Does better engagement improve clinical outcomes?

Engagement requires effort and has inherent limits for each individual. In DMHIs, this challenge is further compounded by the conditions they aim to address - many mental health disorders include low motivation and impaired concentration as core symptoms, potentially hindering sustained engagement in diverse and unpredictable ways^1^. In contrast, digital approaches could have advantages for some specific mental health disorders. For example, virtual reality (VR) exposure therapy for anxiety might be expected to improve treatment retention. In fact, study results have been equivocal, with similar attrition rates between VR and in-vivo exposure treatments^13^, although the data are difficult to assess as the original studies often did not report reasons for dropout and used now-obsolete technology.

In general, attrition has not been widely studied. Factors which have been identified as being involved in early engagement relate more strongly to perceived rather than objective need, and reasons such as forgetting, not finding time, or not finding the digital intervention useful have been associated with attrition^14^. Additionally, attrition rates are likely to vary by mental health condition, treatment type and stage of illness. However, attrition, as well as a marker of loss of engagement, can also be a marker of treatment success. There are positive reasons for disengagement: the participant may have internalised their learning and be using this outside the digital space, or may be using other resources, or have achieved recovery^5,14^. Non-adherence or attrition may also reflect ‘e-attainment’—the discontinuation of engagement because personal goals have been met^15^. In addition, there is no agreed approach for how to assess users who are non-engaged but stay in the study. Effective use patterns may differ from user to user^15^. For example, Chien et al.^16^ identified 5 discrete subtypes of users based on engagement and found that the level of engagement was not always proportional to the observed clinical improvements^16^.

Although it is widely accepted that engagement with digital interventions should be positively associated with improvements in mental health, this has been difficult to demonstrate robustly^17^. Usage is often reported as an outcome in itself, but whilst some usage is needed, there has been little research on what may be the optimal or ‘target’ dose to achieve effective outcomes. For example, a systematic review of DMHIs suggested that greater usage may be correlated with improvements in mental health^18^, but the interpretation was limited as this was measured differently in the different studies. In a review focussing on digital interventions in depression, a small number of studies (14) measured the relationship between usage metrics and outcomes, and of these 9 found an association between increased engagement and improved participant outcomes^8^. In contrast, in some conditions, such as post-traumatic stress disorder (PTSD), ultra-brief treatments have been effective and acceptable^19,20^, challenging the idea that longer engagement or usage are always required. Whilst the usage metrics reported in some research studies may appear promising, this may not always translate to everyday or clinical settings. For example, a review of unguided e-mental health interventions showed that in research studies which proactively recruited users, the median programme usage rate was 4.06 times higher than the subsequent real-world usage^21^. This may be related to the additional factors in trial settings (such as frequent human contact and extra assessments), which are less evident in real-world use. The mechanisms of engagement, including the essential elements or markers of effective engagement have not been clearly identified^3^. Engagement is a complex behaviour, usually starting with a prompt or an interest in adopting an intervention (for example, from a clinician, peer or social media) followed by initial use, and engagement. Disengagement and reengagement with the same or different intervention may also follow^22^. These stages vary in order and time course between individuals, between interventions and during the course of the intervention itself. Engagement strategies may only be effective at some stages—for example novelty may be helpful in initial signing up^14,23^ and habit formation may be more important in sustained use^24^.

### User involvement in developing and delivering DMHIs

The consensus meeting agreed that involving end users in the design and delivery of digital interventions would be expected to enhance engagement and therefore, improve outcomes^25,26^. There are a variety of different approaches to involving the user (for example, co-production, co-design and human or user-centred design) and there has recently been increased interest in user-centred design in digital approaches^27,28^. However, user-centred design approaches are themselves often poorly defined and in practice user involvement in developing and delivering digital health interventions, including DMHIs is variable and often limited to the early and/or final stages of design development and delivery^25,29,30^. In addition, reporting is variable, making assessments or comparisons of user involvement very challenging^25^.

In digital mental health, there are only a few examples of true user-centred co-design. For example, a mapping review of studies in e-mental health interventions focussed on those where they identified user-centred design in their methodology. The papers were then analysed using the steps defined in the UK Design Council’s framework for innovation (https://www.designcouncil.org.uk/our-work/news-opinion/double-diamond-universally-accepted-depiction-design-process/) and from this a variety of approaches were identified. Only 16 studies provided a definition of their chosen design approach and only 5 out of the 27 could be classified as using user-centred design^30^.

## Discussion

The consensus meeting identified a number of potential solutions to these challenges in the area of engagement. These cut across several different challenges and are organised below into broad themes and summarised in Table 2.

**Table 2:** Potential solutions to the challenges of engagement in digital mental health interventions identified by the consensus meeting

*Theme 1 – definitions and terminology* a) **Standardisation of reporting of engagement in DHI research studies**. • The group did not achieve consensus on the exact definitions of each engagement term, but agreed on core concepts • Engagement is a complex term encompassing usage and adherence, but also cognitive, affective and motivational components • Agreed guidance needs to be developed, standardised and implemented by all studies • More than one engagement statistic should be reported (including both objective and subjective measures) • Transparent reporting of raw data is needed to allow direct comparisons **b) Assessment of the appropriate ‘dose’ of an intervention, to maximise engagement and outcomes**. • Short or ultra-short interventions may be appropriate in some cases • The target dose needs to be assessed for each intervention
*Theme 2 - demonstrating efficacy (outcomes) and cost effectiveness of effective engagement* **a) Research studies need to be theory driven**. **b) Research studies should actively report engagement and outcomes**. • Design trials to determine engagement, efficacy/outcomes and their facilitators • It is key to measure both patient-reported outcomes and experiences (PROMS and PREMS) as well as standard outcome measures • More research is needed on the links between engagement and outcomes, including dose relationships and potential (bio)markers for optimal engagement and response.
*Theme 3 – user involvement and user centred design* **a) Improve standards of user involvement in DHI research studies, with more precise reporting**. • Standardised guidance • Co-production and human-centred co-design • User involvement and engagement **b) Investigate the mechanisms of engagement to identify the essential elements**. • Identifying the relative contribution of different engagement strategies • Maximising theory driven work in trustworthiness and engagement **c) Measure and report the potential harms of engagement**. **d) Include clinicians and the wider workforce as users**.

The group did not aim to achieve a consensus on definitions of each term but agreed on the core concepts (Table 2, Theme 1a). Engagement is a complex term encompassing usage and adherence, but also cognitive, affective and motivational components. The group recognised the variety of different definitions and approaches which have already been proposed to describe engagement metrics, including conceptual frameworks for understanding in-the-moment engagement and how this could be used in designing strategies to promote engagement^7^. Engagement can also be conceptualised and measured at the micro level (moment to moment usage and the user experience) and the macro level (including the depth of engagement with the behaviour change process)^31^. These variations in definitions, as well as creating difficulties in comparisons across studies, may also explain at least some of the variability in the rates of engagement reported.

There was also consensus that agreed reporting standards are needed to allow comparison and synthesis of data across all individual research studies. The CONSORT-ehealth guidelines already include subitems relating to reporting attrition and engagement^32^. However, reporting in individual studies still varies extensively^3^ and agreed guidance needs to be developed, standardised and implemented. An important part of these standards would be to report more than one engagement measure, including both objective (usage) and subjective metrics. Usage results should include raw data to allow direct comparisons, and other usage metrics (such as measures of adherence) should be reported transparently, including the pre-specified threshold used and justification for the rationale^11^. Subjective measures of engagement allow convergent evidence to be compared to the behavioural data and should include not only commonly used questionnaires and self-report measures, but also newer metrics such as the Digital Working Alliance Inventory (D-WAI^33^). This assesses the degree of alliance a user has to an app and has been shown to be associated with subjective and objective measures of app engagement and outcomes. Even with standardisation of metrics, however, individual study characteristics reporting will be essential: differing designs of interventions may mean that engagement can be measured only in certain ways in particular studies and the degree of personalisation may make defining the optimal dose more challenging.

Whilst sustained engagement may be needed for some interventions, longer engagement is not necessarily always better, and shorter interventions may be appropriate in some cases (e.g., single session interventions) (Table 2, Theme 1b). Researchers need to consider whether increased and sustained engagement is always required, by assessing the ‘target dose’. For some interventions, ultra-brief treatments may be possible. For example, in a study of an ultra-brief online treatment, self-reported anxiety and depression significantly reduced and the ultra-brief treatment was assessed as non-inferior to a standard-length treatment^34^. Similarly, a guided single-session online intervention was shown to be effective in reducing intrusive memories of work‑related trauma^19,20^. Treatments could be shortened by only focussing on one aspect of a mental disorder (in this example by a focus on reducing intrusive memories in PTSD, rather than the whole symptomatology). This may not be suitable for all mental health conditions, but in some areas research could examine the potential to design shorter courses of digital treatments; rather than focussing on increasing usage it may be better in these cases to focus research efforts on decreasing the need for longer term usage and adherence.

Theory and rationale should direct both the research questions posed and the variables measured, guiding the direction and scope of enquiry (Table 2, Theme 2a). There was consensus that digital research, like its non-digital counterpart, should be guided by clear mechanistic or theoretical rationales. For example, studies should target specific psychological mechanisms with measurable outcomes, such as belief change, and these rationales should be explicitly reported to ensure transparency and scientific rigour^35^. If the proposed theory is explicitly stated and the (bio)markers of interest are identified, this would enable comparison of individual studies. In addition, DHIs present an opportunity to design for more consistent targeting of causal mechanisms and the use of novel features to promote engagement with the mechanisms of change. For example, SloMo is a digitally supported psychological therapy for paranoia which supports visualisation of thinking habits (the key mechanism of change) as bubbles, which provide an engaging means of communicating subjective experiences while reducing information processing demands^27,35,36^. Rather than simply adapting traditional therapy approaches into a digital format, mechanistic work is needed to identify the key processes in disorders in order to inform hypothesis-driven research in developing new and engaging DMHIs^37^.

Trial design in digital health should be optimised to actively assess engagement, outcomes and their facilitators (Table 2, Theme 2b). Engagement with digital health tools and interventions is a complex behaviour which can be affected by internal facilitators (such as technology, gamification, design features) and external facilitators (such as using a digital navigator and a blended approach within a digital clinic environment)^38^. Engagement can also be influenced by tailoring the intervention to a particular disease area or patient characteristics. For example, adapting an internet-based cognitive behavioural intervention to the needs of patients with depression in the context of multiple sclerosis (MS) is valued by patients and may increase usage and efficacy^39^ compared to a previous version that did not consider MS specific aspects. A recent study also investigated the impact of digital phenotyping to personalise app recommendation and suggested this resulted in increased engagement as measured by objective screen time and a measure of alliance, but more mechanistic studies are needed^24^. Although common approaches to increase engagement include strategies such as personalisation and customisation, social and therapeutic support, within intervention guidance and real-time feedback^23^, it is not clear which of these contribute most effectively to increased engagement and improved outcomes. Implementing some or all of these will increase costs and in-person time, thus potentially reducing some of the advantages of digital approaches over in-person consultation.

In terms of outcomes, it is key to measure both PROMS (patient reported outcome measures, for example symptoms, daily activity, quality of life) and PREMS (patient reported experience measures such as satisfaction, communication/shared decision making, health literacy, autonomy and ease of access to healthcare) as well as standard objective measures of clinical outcomes, such as morbidity, mortality, or disease duration. Digital solutions are ideally suited to collect these and feed them back to the clinician and patient, for example, in cancer care^40^ or in mood disorders^41^. Patients’ ‘empowerment’ (measures of the real-world outcomes of engagement) should also be reported across a variety of domains such as emotional and social wellbeing, self-management and control, education and knowledge, including health literacy and engagement in healthcare^42^. Research should focus on the links between engagement, usage and outcomes, not only looking at ‘dose’ effects, but also the potential to identify markers for response from the early sessions. This will also rely on standardising reporting of engagement metrics (Theme 1).

There was consensus that PPIE should be intentional and extend beyond a consultation model (Table 2, Theme 3a). The goal of PPIE is to ensure that DHIs meet the essential rights of users to be included in the development of relevant interventions and to ensure these are appropriately focussed on delivering real-world benefits for that particular population. Patients bring lived expertise, offering unique insights into their condition and the challenges of digital tools that may be overlooked by others. Representation from the full diversity of the target population aims to increase not just usage but also meaningful engagement and outcomes^25,43^ and attempts to mitigate at least some of the impacts of the digital divide^44^. Diversity includes adequate representation from underserved populations (e.g., older adults, individuals with lower digital literacy, from disadvantaged socioeconomic backgrounds or ethnic minorities) as well as those at the intersection of these categories. The following areas of focus were identified:

- Standardised guidance: one barrier to greater user involvement may be that for digital approaches, including app design, there is, as yet, no standardised guidance on how to involve stakeholders, although frameworks have been proposed^25^. There are frameworks for user involvement more generally, such as the UK National Institute for Health and Care Research (NIHR) INVOLVE framework (https://www.nihr.ac.uk/news/nihr-announces-new-standards-public-involvement-research), and the UK Design Council’s Double Diamond model (https://www.designcouncil.org.uk/our-resources/the-double-diamond/), but focused standardised guidance for designing and reporting user input in DHIs is needed.
- Co-production and human-centred co-design: to ensure PPIE is meaningful, a co-production model is often used in clinical research. Although co-production indicates involvement, care needs to be taken as this approach typically focuses on developing and refining previously identified solutions to a previously agreed problem^26^. Whilst this can be helpful, an inclusive human centred co-design approach, including divergent and convergent phases in the development of DMHIs (https://www.designcouncil.org.uk/our-resources/the-double-diamond/), may be needed to facilitate more effective engagement across diverse users. This method is distinct from coproduction in that it uses ethnographic methods to explore the needs and preferences of a diverse range of people, with iterative co-design of solutions to address these identified needs. The process aims to investigate ‘what people need, rather than what they say they want’^26^. This process aims to optimize the user experience and improve adherence for a diverse range of people^45^ and involves a significant input of time, planning and funding, with only a few examples in mental healthcare so far^27^. Whilst user-centred design may improve engagement^28^, more direct evidence is needed. This might take the form of direct comparisons of different versions of DHIs or mechanistic studies to investigate key elements of engagement contributing to improved outcome. To assess how user centred co-design contributes to improved engagement and outcomes, studies need to report transparently the types of PPIE, who is involved and at what stage of product development with clearer definitions of the exact methodology used. Reporting of diversity among stakeholders is also a challenge, and studies should include and report involvement of both expert PPIE and inclusive, diverse PPIE^25^. PPIE models should be carefully planned with sufficient allocated resources in terms of time and funding^25^. It is essential to investigate the relationship between different types of PPIE and outcomes, as the investment of time and resources could offset some of the potential advantages of digital approaches.
- User involvement and engagement: user involvement also needs to focus on meaningful engagement and outcomes (not just increased usage). This should include recognising the ‘engagement paradox’ and designing for disengagement once participants have reached their personal goals. Users may disengage for positive as well as negative reasons—these need to be tracked and reported in studies. Digital approaches have the potential to address some of the existing inequalities in care provision and to increase engagement in underserved populations, but to do this effectively they need to be proactively designed to mitigate issues exacerbating the digital divide^44,45^.

Mechanistic work is required, focussing specifically on the mechanisms underlying engagement and its translation to optimal outcomes, and whether there are identifiable (bio)markers for these (Table 2, Theme 3b). For example, some research suggests that working alliance and self-efficacy may be potential mediators between engagement and outcomes^46^. Digital interventions offer advantages in terms of scalability, personalisation and integrated measurement of usage, but these can only be maximised if the elements which are essential for successful engagement are identified. Researchers need to consider what are the best approaches to study the mechanism of engagement in DMHIs, such as platform trials or Bayesian approaches and the use of analytic approaches such as machine learning and artificial intelligence (AI)^47^. Frameworks such as the technology acceptance model (https://deepblue.lib.umich.edu/handle/2027.42/35547) can help identify key factors using broad categories of perceived usefulness, perceived ease of use, and actual use behaviour as relevant categories in determining engagement.

Mechanistic studies of engagement would allow the identification of the approaches which specifically improve engagement and outcomes. Strategies such as frequent contact, personalised feedback, gamification, and financial incentives can help reduce attrition rates^1,48^, and integrating these within digital interventions through automated notifications, prompts, and feedback has also shown promise^3,49,50^. The digital space is also unique in that tools such as gamification, as well as increasing engagement, can also be a part of the therapy in themselves. For example, in gameChange, a VR therapy for agoraphobic avoidance in psychosis^51^, users play a bubble-popping game in a virtual café. This allows users to test their fears about other people while building positive memories of social situations. Peer and therapist support (such as a ‘peer digital navigator’^52^) for digital interventions can also promote better engagement. Support through social networks can increase engagement^9^ and may also have a positive effect on symptoms (for example, of depression)^41^, although the exact mechanisms and essential ingredients are not fully elucidated. Personalisation of interventions can occur at several different levels to promote greater engagement and inclusivity: the DHI can be personalised and adapted to individual preferences and characteristics (within the technical specification), or more generally tailored according to their disease area, symptoms or age^53^. For example, in DMHIs, mental health symptoms such as low motivation and impaired concentration as core features of anxiety and depressive disorders may directly affect engagement, as well as physical comorbidities such as fatigue, pain and sensory impairment^41^. In addition, there can be personalisation in identifying which DHI is the correct fit for the individual (which may be facilitated by a staff member such as a ‘digital navigator’)^24^. It is likely that a combination of different factors may be needed for a specific intervention or disease area. For example, in a scoping review of apps for schizophrenia, strategies used to improve engagement included push notifications and message prompts, personalisation, goal setting, gamification, multimedia formats, social connectedness, and support (peers and professionals)^5^, but the individual contribution of each strategy and their relative contributions has not yet been assessed.

It is also important to consider how to maximise the perceived ‘trustworthiness’ with DHIs, by harnessing the intrinsic benefits of what is already known about participant empathy and therapeutic relationships in the digital space. For example, there is a well-researched tendency for humans to anthropomorphise inanimate objects, which extends to digital interactions, including chatbots and conversational agents. Whereas online surveys show that a significant number of patients delay in person help-seeking because of embarrassment or a fear of being judged^54^, rates of consultations with online platforms are high. For example, in a user survey of 2000 US adults, 67% of Americans said they had looked up their symptoms on an internet search engine and 52% had used a large language model like ChatGPT, looking for a diagnosis (https://www.usertesting.com/resources/reports/consumer-perceptions-ai-healthcare). Transdisciplinary expertise in digital empathy is needed to maximise effective engagement – a degree of humanising the interface can improve engagement, but if ‘too human’ this may discourage disclosure of negative or embarrassing information^55,56^.

It is essential to measure and report potential adverse events actively in studies as they could also be a significant reason for dropout and loss of engagement^57^ (Table 2, Theme 3c). Adverse events could range from mild effects (such as frustration, boredom) to more severe (for example, symptom deterioration, suicidality or hospital admission). Their severity can also depend on the perceived impact on the patient; for example, screen time is often perceived as a potential adverse event, whereas the impact may be more nuanced and depend on the individual context (https://www.mqmentalhealth.org/mental-health-and-the-internet/).

Just as participant user involvement should increase engagement, clinicians can also influence the adoption of digital interventions (Table 2, Theme 3d). Clinicians vary in their confidence and experience in the use of DMHIs. Increasing these would involve education and training in digital approaches^58^ and involving a diverse range of clinicians as ‘experts by experience’ at an early stage would facilitate better integration of DMHIs into the clinical pathway. Frameworks such as the Non-adoption, Abandonment, and challenges to Scale-up, Spread, and Sustainability (NASSS) framework^59^ can provide a structured approach for staff involvement, helping identify barriers and facilitators to real-world implementation throughout DMHI development and deployment. For example, the NASSS framework has been used to identify NHS staff views on the implementation of VR interventions on acute psychiatric wards, identifying both challenges (staff confidence with technology) and potential solutions (such as having a staff VR lead and accessible training)^60^.

In this study we aimed to identify challenges and potential solutions in studying and enhancing digital health engagement, and how this might translate to improved outcomes for users and to focus the field for future research in this area. However, we are aware that there may be some potential limitations. While we conducted a systematic review, we limited our search to PubMed, which may have excluded relevant publications. We are also aware of the potential biases in the ways that industry and academic research sectors report and analyse engagement metrics, which may have affected the results reported^8^. Additionally, like all consensus meetings, ours lacked standardized criteria for defining expertise. Although we selected participants to represent a diverse spectrum of views, the reliability of consensus opinions is dependent on the specialist knowledge and experiences of those who participated. We sought to ensure diverse perspectives by assembling an expert group and panel with varied expertise, nationalities, genders, ages, and disciplinary backgrounds. While the expert group included a wide range of experience in clinical research and real-world implementation in digital mental health, in future studies, we would also consider including contributions from commercial partners. In terms of lived experience, we included an expert with lived experience who made many material contributions throughout: to the literature review, presentation and discussion of the evidence, formation of consensus, and coproduction of the paper. In this way, we aimed to engage high-quality PPI coproduction and we also identified a number of changes for future consensus meetings which could be implemented. For instance, in future meetings we will provide a glossary of terms with acronyms spelt out and lay definitions of scientific terminology to be used before and during the meeting to facilitate equal understanding. In addition, consistent with other examples of lived experience coproduction in mental health research^61^, the digital topic will be chosen in collaboration with lived experience members.

This study utilised an international expert meeting, including lived experience and used a documented consensus method to incorporate the current state of evidence into our discussions. From this, we developed a consensus on the current challenges and next steps for assessing, recording and analysing engagement with DMHIs and their association with outcomes. Digital interventions have exciting potential in meeting the shortfall in service provision for participants with brain and mental health disorders. However, this can only be realised if we focus our efforts on high-quality standardised measures and reporting to identify which factors promote meaningful engagement and lead to more reliable real-world outcomes.

## Methods

We used the consensus development panel (or consensus development conference (CDC)) approach and followed the methodology described and used by the US National Institutes of Health and the World Health Organization (www.who.int/publications/i/item/9789241548960)^62,63^. This is a particularly effective consensus method for identifying areas of challenge and potential solutions in a rapidly developing area and has been used in previous consensus studies in digital health^64,65^. Central to the methodology of the CDC is a face-to-face meeting between a group of individual experts and a separate panel of nonexpert participants, involving an interactive method to develop a consensus. The method enables a multidisciplinary approach, including lived experience, with all group members contributing to the discussion and recommendations and incorporating a literature review of the existing evidence.

### The consensus meeting

The in-person meeting was held in Rome over 2 days in November 2024 and involved an international multidisciplinary group of individual experts (including with lived experience of mental health issues) and a separate panel of nonexpert participants (hereafter, “the panel”). In advance of the meeting, the panel conducted a systematic literature review using PubMed to search for papers relevant to the main themes identified by the experts (see Supplementary Note 1 for further details). This preliminary work formed the agenda for the questions to be addressed during the meeting. Consensus was defined as either fully met or unmet, with the outcome transparently reported^66^. At the end of the meeting, the whole group engaged in plenary discussion to identify the key themes and structure the recommendations. The group identified challenges, which are outlined in the results, and potential solutions in the discussion.

### The expert group

The 10 experts (AC, CB, SMG, EH, JK, S. Lambe, JT, PU, TW, AV) encompassed expertise in a variety of specialist areas within digital health (including virtual reality, coproduction and co-design, web-based screening and early intervention, digital approaches to empathy and the therapeutic relationship, philosophy, ethical issues, and lived experience). The group composition was gender-balanced, and professional backgrounds and experience included psychiatry, neurology, psychology, cognitive neuroscience, social sciences, methodology, evidence synthesis, regulatory pathways, patient and public involvement (PPI), philosophy and ethics. The expert group was international (including members from Germany, Luxembourg, Sweden, the United Kingdom, and the United States).

### The panel

The panel was composed of 10 members (KB, TG, CGS, KH, S. Lahutina, EGO, IP, SR, KAS, LT) and included early-career and more experienced clinicians and researchers (at a different level of expertise). The panel members were chosen because they were well informed or experienced in mental health and digital interventions, but had no particular expertise in any one area of digital psychiatry. The panel was also international (including members from Germany, Italy, Luxembourg, Mexico, Sweden, Turkey, the United Kingdom, and the United States).

The panel increased their knowledge of the current evidence base in advance of the meeting by conducting a systematic literature review using PubMed to search for terms relevant to the main themes identified by the experts (see Supplementary Note 1 for further details). This preliminary work highlighted the areas of recent development, uncertainties, or challenges that formed the agenda for the questions to be addressed in the face-to-face meeting. Of the 261 papers identified by the panel in the systematic search, 11 (narrative and systematic reviews) were identified as essential reading (see Supplementary Fig. 1 for further details). All panellists were asked to read the selected papers and panel members were allocated to lead the group discussion on one expert talk to facilitate equal contributions from members of the expert group and the panel.

### Reflexivity statement

The meeting was convened by JT and AC, who selected the expert group to represent a balance of professional backgrounds, areas of specialist digital mental health expertise, lived experience, and gender. Panel members were suggested by members of the expert group and through professional contacts. The logistics of the meeting were supported externally by Angelini Pharma, but they did not have any input in the design of the meeting, identification or selection of the expert group or panel, agenda of the meeting, discussions, consensus, or output. Ethical approval was not required for this study as it did not involve research on human participants. The consensus was conference based, and all attendees offered contributions to the research topic in an open environment where talks were voluntary.

## Supplementary information

Supplementary information

## Data Availability

All data generated or analysed during this study are included in the published article and the Supplementary Information.
