# Supplementary material for: Engagement and attrition in digital mental health: current challenges and potential solutions
Source: NPJ Digit Med. 2025 Jul 2;8:398. doi: 10.1038/s41746-025-01778-w (PMC12223045; doi:10.1038/s41746-025-01778-w)
Supplement: Supplementary file 1 — Supplementary information [file 41746_2025_1778_MOESM1_ESM.pdf]

SUPPLEMENTARY INFORMATION

Engagement and attrition in digital mental health: current challenges and potential solutions

Contents

Supplementary Note 1. Systematic literature review and search strategy for systematic review……….2

Supplementary Figure 1. PRISMA flow diagram ………………………………………………………………………………..3

Supplementary References……..4

Supplementary Table 1. Prisma 2020 Checklist………………………………………………………………………………6

Supplementary Note 1. Systematic literature review and search strategy for systematic review

We performed a literature review of the records published from inception on PubMed focussing on engagement in digital health.

We used a broad search strategy and the search was performed on 28th August 2024 using the following search strategy:

#1 "Digital Health"[MeSH Terms] OR "Telemedicine"[MeSH Terms] OR "digital"[Title] OR "wearable"[Title/Abstract]

#2 "Mental Health"[MeSH Terms] OR "Psychiatry and Psychology Category"[MeSH Terms]

#3 "engagement"[Title/Abstract] OR "adherence"[Title/Abstract]

#4 #1 AND #2 AND #3

The screening process was completed independently by the ten panellists using Rayyan, a web-based and mobile app automated screening tool[1]. Any queries were resolved by team discussion. Papers were eligible for inclusion if they were relevant to engagement and digital health. The search resulted in 3696 records, 3250 of which were excluded after a title/abstract screening (please see further details of the search in Supplementary Figure 1).

At the full-text screening, 446 records met the inclusion criteria. Of these, 11 were selected as essential reading by the panel and shared with all the panellists. Each expert suggested up to 5 relevant references for their talk and these were also shared with the panel.

Supplementary Figure 1. PRISMA (Preferred Reporting Items for Systematic Reviews and Meta-Analyses[2]) flow diagram

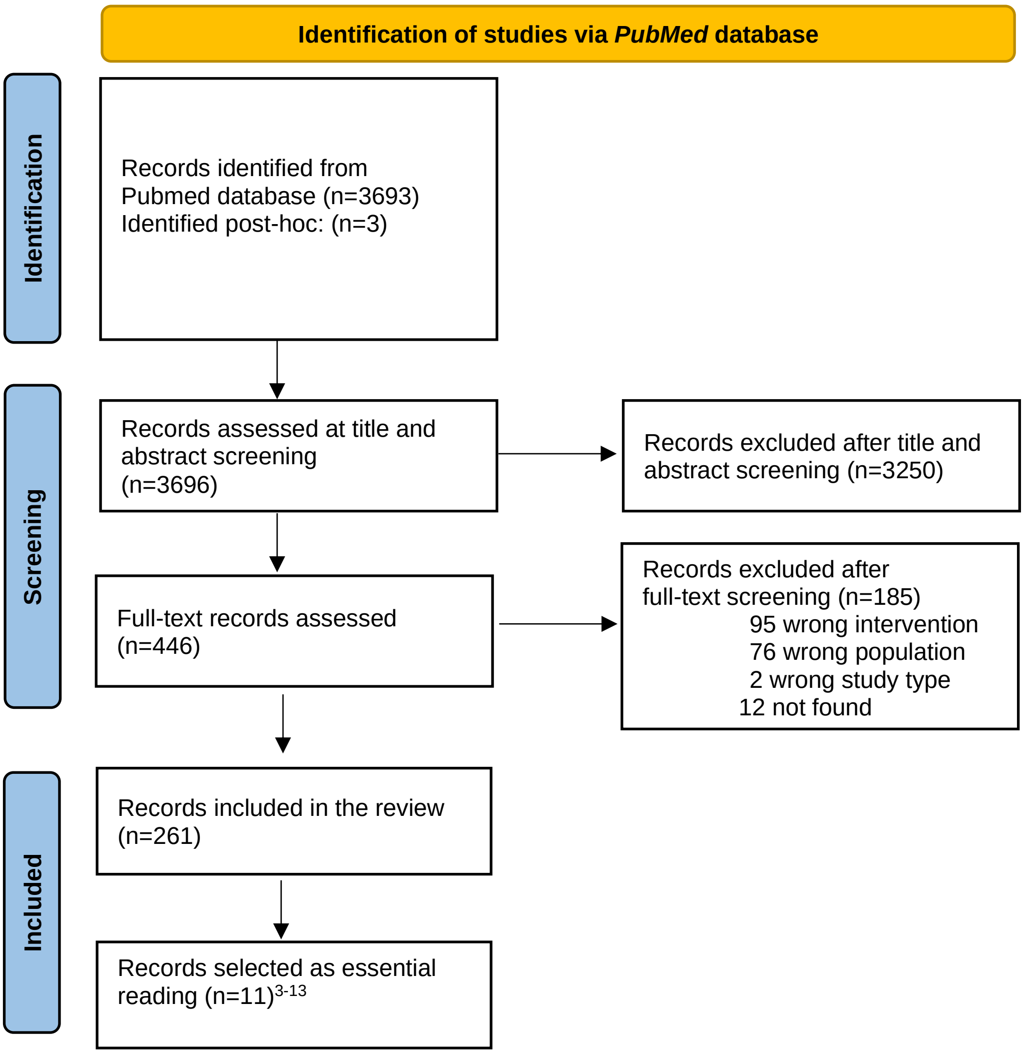

Supplementary References

Additional references (included here only those references not in the main paper)

1. Ouzzani, M., Hammady, H., Fedorowicz, Z., & Elmagarmid, A. (2016). Rayyan—a web and mobile app for systematic reviews. *Systematic reviews*, **5,** 1-10 (2016).
2. Page, M. J., et al. PRISMA 2020 explanation and elaboration: updated guidance and exemplars for reporting systematic reviews. *Bmj*, **372** (2021).

Papers included in the review as essential reading

3. Borghouts, J., et al. Barriers to and facilitators of user engagement with digital mental health interventions: systematic review. *Journal of medical Internet research*, ***23*,** e24387 (2021).
4. Elkes, J., et al. User engagement in clinical trials of digital mental health interventions: a systematic review. *BMC medical research methodology*, **24,** 184 (2024).
5. Forbes, A., Keleher, M. R., Venditto, M., & DiBiasi, F. Assessing patient adherence to and engagement with digital interventions for depression in clinical trials: systematic literature review. *Journal of Medical Internet Research*, **25,** e43727 (2023).
6. Gan, D. Z., McGillivray, L., Han, J., Christensen, H., & Torok, M. Effect of engagement with digital interventions on mental health outcomes: a systematic review and meta-analysis. *Frontiers in digital health*, **3,** 764079 (2021).
7. Gan, D. Z., McGillivray, L., Larsen, M. E., Christensen, H., & Torok, M. Technology-supported strategies for promoting user engagement with digital mental health interventions: A systematic review. *Digital health*, **8,** 20552076221098268 (2022).
8. Nahum-Shani, I., Shaw, S. D., Carpenter, S. M., Murphy, S. A., & Yoon, C. Engagement in digital interventions. *American Psychologist*, **77,** 836 (2022).
9. Perret, S., et al. Standardising the role of a digital navigator in behavioural health: a systematic review. *The lancet digital health*, **5,** e925-e932 (2023).
10. Short, C. E., et al. Measuring engagement in eHealth and mHealth behavior change interventions: viewpoint of methodologies. *Journal of medical Internet research*, **20,** e292 (2018).
11. Sieverink, F., Kelders, S. M., & van Gemert-Pijnen, J. E. Clarifying the concept of adherence to eHealth technology: systematic review on when usage becomes adherence. *Journal of medical Internet research*, **19,** e402 (2017).
12. Smith, K. A., et al. Digital mental health for schizophrenia and other severe mental illnesses: an international consensus on current challenges and potential solutions. *JMIR Mental Health*, **11,** e57155 (2024).
13. Yardley, L., et al. Understanding and promoting effective engagement with digital behavior change interventions. *American journal of preventive medicine*, **51,** 833-842 (2016).

Supplementary Table 1. Prisma 2020 Checklist

Section and Topic	Item #	Checklist item	Location where item is reported
**TITLE - Assessing the impact of evidence-based mental health guidance in COVID-19: a systematic review and qualitative evaluation**			Page/section
Title	1	Identify the report as a systematic review.	P3 (Abstract), P19-20 (Methods), P17-18 (Discussion), Supplementary Note 1
**ABSTRACT**			
Abstract	2	See the PRISMA 2020 for Abstracts checklist.	P3 (Abstract)
**INTRODUCTION**			
Rationale	3	Describe the rationale for the review in the context of existing knowledge.	P4 (Introduction)
Objectives	4	Provide an explicit statement of the objective(s) or question(s) the review addresses.	P4 (Introduction)
**METHODS**			
Eligibility criteria	5	Specify the inclusion and exclusion criteria for the review and how studies were grouped for the syntheses.	Supplementary Note 1
Information sources	6	Specify all databases, registers, websites, organisations, reference lists and other sources searched or consulted to identify studies. Specify the date when each source was last searched or consulted.	Supplementary Note 1
Search strategy	7	Present the full search strategies for all databases, registers and websites, including any filters and limits used.	Supplementary Note 1
Selection process	8	Specify the methods used to decide whether a study met the inclusion criteria of the review, including how many reviewers screened each record and each report retrieved, whether they worked independently, and if applicable, details of automation tools used in the process.	Supplementary Note 1
Data collection process	9	Specify the methods used to collect data from reports, including how many reviewers collected data from each report, whether they worked independently, any processes for obtaining or confirming data from study investigators, and if applicable, details of automation tools used in the process.	Supplementary Note 1
Data items	10a	List and define all outcomes for which data were sought. Specify whether all results that were compatible with each outcome domain in each study were sought (e.g. for all measures, time points, analyses), and if not, the methods used to decide which results to collect.	Not applicable (search was used to inform panel members)
	10b	List and define all other variables for which data were sought (e.g. participant and intervention characteristics, funding sources). Describe any assumptions made about any missing or unclear information.	Not applicable
Study risk of bias assessment	11	Specify the methods used to assess risk of bias in the included studies, including details of the tool(s) used, how many reviewers assessed each study and whether they worked independently, and if applicable, details of automation tools used in the process.	Not applicable
Effect measures	12	Specify for each outcome the effect measure(s) (e.g. risk ratio, mean difference) used in the synthesis or presentation of results.	Not applicable
Synthesis	13a	Describe the processes used to decide which studies were eligible for each synthesis (e.g. tabulating the	Not applicable

Section and Topic	Item #	Checklist item	Location where item is reported
methods		study intervention characteristics and comparing against the planned groups for each synthesis (item #5)).	
	13b	Describe any methods required to prepare the data for presentation or synthesis, such as handling of missing summary statistics, or data conversions.	Not applicable
	13c	Describe any methods used to tabulate or visually display results of individual studies and syntheses.	Not applicable
	13d	Describe any methods used to synthesize results and provide a rationale for the choice(s). If meta-analysis was performed, describe the model(s), method(s) to identify the presence and extent of statistical heterogeneity, and software package(s) used.	Not applicable
	13e	Describe any methods used to explore possible causes of heterogeneity among study results (e.g. subgroup analysis, meta-regression).	Not applicable
	13f	Describe any sensitivity analyses conducted to assess robustness of the synthesized results.	Not applicable
Reporting bias assessment	14	Describe any methods used to assess risk of bias due to missing results in a synthesis (arising from reporting biases).	Not applicable
Certainty assessment	15	Describe any methods used to assess certainty (or confidence) in the body of evidence for an outcome.	Not applicable
**RESULTS**			
Study selection	16a	Describe the results of the search and selection process, from the number of records identified in the search to the number of studies included in the review, ideally using a flow diagram.	Supplementary Figure 1. PRISMA flow diagram
	16b	Cite studies that might appear to meet the inclusion criteria, but which were excluded, and explain why they were excluded.	Supplementary Figure 1. PRISMA flow diagram
Study characteristics	17	Cite each included study and present its characteristics.	Not applicable (search was used to inform panel members)
Risk of bias in studies	18	Present assessments of risk of bias for each included study.	Not applicable
Results of individual studies	19	For all outcomes, present, for each study: (a) summary statistics for each group (where appropriate) and (b) an effect estimate and its precision (e.g. confidence/credible interval), ideally using structured tables or plots.	Not applicable
Results of syntheses	20a	For each synthesis, briefly summarise the characteristics and risk of bias among contributing studies.	Not applicable
	20b	Present results of all statistical syntheses conducted. If meta-analysis was done, present for each the summary estimate and its precision (e.g. confidence/credible interval) and measures of statistical heterogeneity. If comparing groups, describe the direction of the effect.	Not applicable
	20c	Present results of all investigations of possible causes of heterogeneity among study results.	Not applicable
	20d	Present results of all sensitivity analyses conducted to assess the robustness of the synthesized results.	Not applicable
Reporting biases	21	Present assessments of risk of bias due to missing results (arising from reporting biases) for each synthesis assessed.	Not applicable
Certainty of evidence	22	Present assessments of certainty (or confidence) in the body of evidence for each outcome assessed.	Not applicable
**DISCUSSION**			

Section and Topic	Item #	Checklist item	Location where item is reported
Discussion	23a	Provide a general interpretation of the results in the context of other evidence.	Not applicable
	23b	Discuss any limitations of the evidence included in the review.	P18 (Discussion)
	23c	Discuss any limitations of the review processes used.	P18 (Discussion)
	23d	Discuss implications of the results for practice, policy, and future research.	Not applicable
**OTHER INFORMATION**			
Registration and protocol	24a	Provide registration information for the review, including register name and registration number, or state that the review was not registered.	Not applicable (search strategy is defined in Supplementary Note 1 but this was an internal review to update the panel, so detailed protocol not required)
	24b	Indicate where the review protocol can be accessed, or state that a protocol was not prepared.	Not applicable (see previous response)
	24c	Describe and explain any amendments to information provided at registration or in the protocol.	Not applicable
Support	25	Describe sources of financial or non-financial support for the review, and the role of the funders or sponsors in the review.	Acknowledgments P21
Competing interests	26	Declare any competing interests of review authors.	Competing interests P22
Availability of data, code and other materials	27	Report which of the following are publicly available and where they can be found: template data collection forms; data extracted from included studies; data used for all analyses; analytic code; any other materials used in the review.	Not applicable

From: Page MJ, McKenzie JE, Bossuyt PM, Boutron I, Hoffmann TC, Mulrow CD, et al. The PRISMA 2020 statement: an updated guideline for reporting systematic reviews. BMJ 2021;372:n71. doi: 10.1136/bmj.n71

For more information, visit: http://www.prisma-statement.org/
